# A Delphi study of rescue and clinical subject matter experts on the extrication of patients following a motor vehicle collision

**DOI:** 10.1186/s13049-022-01029-x

**Published:** 2022-06-20

**Authors:** Tim Nutbeam, Rob Fenwick, Jason E. Smith, Mike Dayson, Brian Carlin, Mark Wilson, Lee Wallis, Willem Stassen

**Affiliations:** 1 Emergency Department, University Hospitals Plymouth NHS Trust, Plymouth, UK; 2 Devon Air Ambulance Trust, Exeter, UK; 3 Division of Emergency Medicine, University of Cape Town, Cape Town, South Africa; 4 Emergency Department, Wrexham Maelor Hospital, Wrexham, UK; 5 Academic Department of Military Emergency Medicine, Royal Centre for Defence Medicine, Birmingham, UK; 6 Former Fire Officer (Research), National Fire Chiefs Council, Birmingham, UK; 7 Association for Spinal Injury Research, Rehabilitation and Reintegration, Department of Orthopaedics & Musculoskeletal Science, University College London, London, UK; 8 Imperial Neurotrauma Centre, Imperial College, London, UK; 9 Kent, Surrey and Sussex Air Ambulance, Rochester, UK

## Abstract

**Background:**

Approximately 1.3 million people die each year globally as a direct result of motor vehicle collisions (MVCs). Following an MVC some patients will remain trapped in their vehicle; these patients have worse outcomes and may require extrication. Following new evidence, updated multidisciplinary guidance for extrication is needed.

**Methods:**

This Delphi study has been developed, conducted and reported to CREDES standards. A literature review identified areas of expertise and appropriate individuals were recruited to a Steering Group. The Steering Group formulated initial statements for consideration. Stakeholder organisations were invited to identify subject matter experts (SMEs) from a rescue and clinical background (total 60). SMEs participated over three rounds via an online platform. Consensus for agreement / disagreement was set at 70%. At each stage SMEs could offer feedback on, or modification to the statements considered which was reviewed and incorporated into new statements or new supporting information for the following rounds. Stakeholders agreed a set of principles based on the consensus statements on which future guidance should be based.

**Results:**

Sixty SMEs completed Round 1, 53 Round 2 (88%) and 49 Round 3 (82%). Consensus was reached on 91 statements (89 agree, 2 disagree) covering a broad range of domains related to: extrication terminology, extrication goals and approach, self-extrication, disentanglement, clinical care, immobilisation, patient-focused extrication, emergency services call and triage, and audit and research standards. Thirty-three statements did not reach consensus.

**Conclusion:**

This study has demonstrated consensus across a large panel of multidisciplinary SMEs on many key areas of extrication and related practice that will provide a key foundation in the development of evidence-based guidance for this subject area.

**Supplementary Information:**

The online version contains supplementary material available at 10.1186/s13049-022-01029-x.

## Background

Approximately 1.3 million people die each year globally as a direct result of motor vehicle collisions (MVCs) [1]. Following a MVC some patients will remain trapped in their vehicle; these patients have worse injuries and are more likely to die than their untrapped counterparts [2]. Patients who are trapped may require assistance in leaving their vehicle; this assistance is termed ‘extrication’ and is often delivered by the rescue services [3]. Extrication may be simple, such as releasing a stuck door, or complex, with specifically designed tools and techniques being used to alter the internal and external structures of the vehicle [3].

The current standard approach to extrication prioritises absolute movement minimisation which contributes to prolonged extrication times [4–6]. Such ‘traditional’ approaches to extrication have recently been challenged by evidence demonstrating the relative rarity of unstable spinal injury or spinal cord injury compared to other time-critical injuries[2]. In addition biomechanical studies in healthy volunteers have demonstrated that rescue service extrication techniques cause more movement than self-extrication, further questioning the accepted approach to extrication [7–9].

Given this new evidence, we need to reconsider the current approach to extrication. The evidence base is wide and diverse, including a large variety of experimental techniques from a broad range of disciplines. These approaches and disciplines include, but are not limited to; rescue service descriptive accounts, biomechanical analyses, clinical case reports, case series, expert opinion, patient experience, crash investigation reports, road safety expert opinion, car design literature and others. A narrative review of this literature is available in the additional file for this paper. The complex nature and wide variety of potential circumstances and subsequent energy transfer that occurs in a MVC, the number, demographics and susceptibility to injury of the patients involved, their injuries and the availability of each aspect of the multi-professional response makes the design and delivery of traditional ‘clinical’ trials in this area an impractical challenge.

The diverse evidence base, requirement for pragmatic expert translation of evidence to practice and the need to achieve multi-professional consensus makes this subject area highly suitable for iterative multi-stage consensus research techniques, such as a Delphi study [10, 11].

The aim of this Delphi study is to develop multi-professional consensus on the evidence-based approach to extrication.

## Methods

**Fig. 1:**
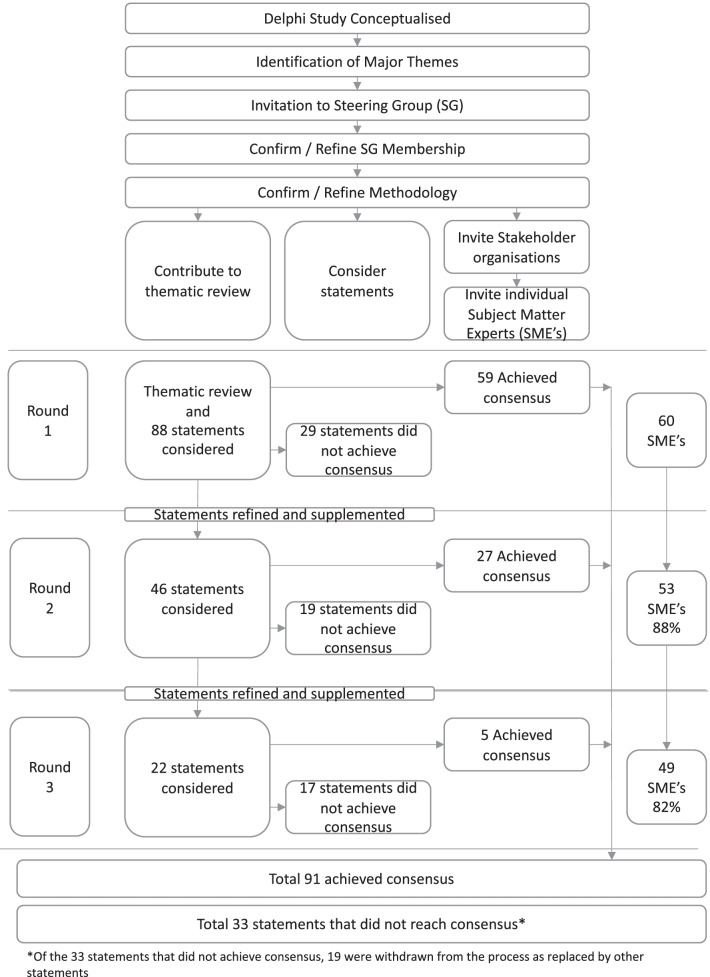
Summary of methods and progression of statements and SMEs through the study

This Delphi study has been developed, administered and reported to the guidance on Conducting and Reporting Delphi Studies (CREDES) standards [10]. The methods are summarised in Fig. 1.

The principal researcher (TN), through a review of the literature identified key areas of expertise that should be represented in a Steering Group for a study in this area of practice. This included individuals with expertise in extrication, prehospital care, trauma care, neurotrauma and representatives of patients with spinal cord injury. Experts with an interest in each of these areas were identified and recruited to offer guidance to the principal researcher within their areas of specialist interest, provide feedback on methodology and process, aid in the production and refinement of statements for the Delphi group and ensure methodological rigour. Joining the steering group excluded an individual as a participant in the Delphi (or subject matter expert, SME).

The Steering Group identified professional organisations that are key stakeholders in UK extrication practice. Stakeholder groups identified were the National Fire Chiefs Council (NFCC), the United Kingdom Rescue Organisation (UKRO), the National HEMS Research & Audit Forum (NHRAF), the College of Paramedics (CoP), the Pre-Hospital Trainee Operated Research Network (PHOTON) and the Faculty of Prehospital Care (FPHC). Each stakeholder organisation was invited to identify up to ten representatives (SMEs). To qualify, SMEs needed to have at least five years of operational experience of delivering extrication or caring for patients during or post entrapment.

Statements for consideration originated from the current evidence base (including unpublished work reporting patient experience) and were proposed by the Steering Group and other stakeholders. All responses were collated and similar statements were collapsed. All materials, including surveys, statements and other written information were reviewed by the Steering Group and subsequently piloted with a multi-professional representative group of SMEs prior to further distribution.

The Delphi was conducted over three rounds, each of which were designed and delivered through the web-based platform Jisc online surveys (JSIC, https://www.onlinesurveys.ac.uk/ 2022). Identified SMEs (60 total) were provided with details of this Delphi study, the statements for consideration, an evidence synthesis (available as supplementary material), an invitation to participate in the study and an online consent form. Throughout the study the anonymity of the SME group was preserved. In each round, SMEs were invited to review the evidence synthesis for each domain of extrication practice and then for each statement using a three-point scale (agree, neither agree nor disagree, disagree) to indicate their opinion. In addition, for each statement the SME had the option to ‘opt out’ if the specific question was outside of their area of expertise. For each statement SMEs had the option to provide free text feedback; including the opportunity to refine current statements and suggest alternative statements for consideration in the following round.

Consistent with previous studies, consensus was set a priori at 70% agreement or disagreement of participating SMEs [12, 13]. Between each round, statements that reached consensus were removed. Statements that did not reach consensus were refined if consistent feedback indicated that this would improve or clarify the statement. Additional suggested statements were collapsed and made available in the following round. If SMEs did not participate in a round they were not eligible to participate in subsequent rounds.

Statements that were accepted or rejected were reported with supporting references. Those statements that reached consensus were summarised and presented individually to stakeholder organisations. Following iterative review an agreed set of clinical and operational principles on which future guidance would be based were agreed by participating stakeholders.

The Faculty Research Ethics and Integrity Committee at the University of Plymouth (ref. 19/20–1313) and the Human Research Ethics Committee at the University of Cape Town (ref. 183/2021) approved the study.

## Results

Rounds 1–3 were conducted in January and February 2022. The background and experience of SMEs are summarised in Table 1.

**Table 1:** Professional, employer and experiential background of SMEs

Demographic	Detail	Number (%)
Professional background	Fire and rescue service	14 (23.3)
	Paramedic	30 (50)
	Doctor	15 (25)
	Nurse	1 (1.7)
Primary employer	Fire and rescue service	14 (23.3)
	Clinical service	45 (75)
	Both	1 (1.7)
Clinical or operational experience	Up to 10 years	19 (31.7)
	11 to 15 years	10 (16.7)
	16 to 20 years	12 (20)
	Over 20 years	19 (31.7)

Figure 1 summarises the study. In Round 1, 88 statements were considered by 60 SMEs. Sixty statements achieved consensus (58 agree, 2 disagree). Free text feedback from SMEs led to three of the original statements from Round 1 progressing to Round 2 for reconsideration (with additional commentary) and the remaining 25 statements were refined and split to make a total of 46 statements presented at Round 2, where 27 statements achieved consensus and 19 did not. Following feedback in Round 2, 22 statements were presented to SMEs in Round 3 of which 5 achieved consensus and 17 did not (Table 2).

**Table 2:** Statements achieving consensus by theme

Theme and Relevant Publications	Statement	Number of SME opt outs
*Terminology* [14–22]	A Multi-Professional Standardised Terminology (MPST) should be developed and adopted to describe different extrication approaches and their variants	0
	The term "patient" is used to refer to the (potentially) injured person post motor vehicle collision regardless of entrapment status	0
	A MPST should be adopted to describe risks and hazards at a scene of an entrapped patient	0
	A MPST should be adopted to described how badly injured and or time-critical entrapped patients are	0
	A MPST should be developed and adopted to describe the entrapment status of patients (e.g. medically trapped, physically trapped)	0
	A MPST should be developed and adopted to describe different extrication techniques as per Joint Emergency Services Interoperability Principles (JESIP)	0
	A MPST should be developed and adopted to describe how rapidly a patient needs to be extricated	0
	Nomenclature for categories of patient: *Not injured* *Minor injuries* (evidence of energy transfer but no evidence of time-dependent injury) *Major injury* (currently stable but should be assumed to be time-dependent) *Time critical injured* (Time critical due to injury; use fastest route of extrication) *Time critical hazard* (Time critical due to a hazard such as fire)	0
*Extrication goals and approach* [14–22]	The historical focus on absolute movement minimisation is no longer justified given information on rarity of spinal injury and frequency of other time critical injuries	0
	The rescuer goal in consideration of patient movement should be “Gentle patient handling”	1
	Minimising entrapment time should be a multi-professional goal for all entrapped patients	0
	Self-extrication or minimally assisted extrication should be the standard ‘first line’ extrication for entrapped patients who are conscious and likely to be able to stand with assistance	0
	Extrication routes (other than self-extrication) appear to be bio-mechanically similar, so it is reasonable to choose the quickest deliverable route given the specific circumstances of the incident	0
	Unconscious patients have high risk of significant injuries and should have an expedited extrication undertaken using ‘gentle patient handling’ techniques	0
	Extrication goals and approach should be similar regardless of the sex or gender of a patient	1
	Patients with acute neurological deficit (e.g. pins and needles in arms) may have time dependent pathology. They should be handled “gently” throughout and entrapment time should be minimised	2
	FRS and clinicians should work together (as per JESIP principles) to plan and deliver a patient and rescuer centred extrication strategy	0
	When environmental conditions permit, FRS personnel should be trained and empowered to plan and complete extrication when clinicians are not available	0
*Self-extrication* [7, 23–31]	All patients should be assessed to see if they are suitable for self-extrication as the primary method of extrication	0
	Patients with neck and / or spinal pain should be considered for self-extrication	3
	Patients with lower limb injuries should be considered for assisted self-extrication	1
	Patients regardless of their injuries should be assessed for suitability for (assisted) self-extrication	1
	Patients with evidence of neurological injury (e.g. pins and needles in arms) may have a spinal cord injury. Patients in this group that can self-extricate, with or without assistance should be encouraged to do so (as this method is associated with smallest movement and shortest entrapment time)	4
	FRS should be trained and empowered to assess patient suitability for self-extrication and assist with this if required	2
	Patients of all ages who are normally mobile should be considered for self-extrication	1
	Patients of all ages should be assessed for actual and potential injuries and a bespoke extrication strategy planned and delivered	1
	Patients with suspected open book pelvic injuries SHOULD NOT be considered for (assisted) self-extrication	5
	Contraindications to self-extrication include: i) an inability to understand or follow instructions, ii) injuries or baseline function that prevents standing on at least one leg, (specific injuries include: unstable pelvic fracture, impalement, bilateral leg fracture)	4
	Patients without contraindications can be considered for self-extrication	3
	Considering statements that define suitability for self-extrication, further consideration of specific pelvic related contraindications are not required	6
*Disentanglement* [3, 5, 7, 16, 19, 26, 32–38]	Patients who are physically entrapped as a result of intrusion have a high likelihood of significant injuries and as such should be considered time critical	1
	Disentanglement should be followed by the quickest appropriate extrication type	2
	Disentanglement should be followed by the quickest appropriate extrication type including self-extrication	2
	Collisions where patients require disentanglement should trigger a senior FRS extrication response	12
	Collisions where patients require disentanglement should trigger an ‘enhanced’ clinical care response^1^	3
	Collisions where patients require disentanglement should trigger a ‘critical-care’ clinical response^2^	4
	Entrapped patients with evidence of energy transfer (injury) should be considered to have time-dependent injuries and entrapment time should be minimised	2
	Collisions where patients require disentanglement are associated with significant injuries to patients, as such FRS should provide an enhanced* response to such incidents. *Accepting that this term and the response will require definition	3
	Post-extrication patients who were entrapped should be carefully and comprehensively assessed, and where appropriate, transferred preferentially to a major trauma centre	1
	Clinical procedures such as intubation and thoracostomy should ideally be delayed until a patient has been extricated	2
*Clinical care* [16, 17, 39–44]	Clinical care should be limited to necessary critical interventions to expedite safe extrication	3
	Rescuers should be aware that clinical observations may prolong entrapment time and as such should be kept to the minimum required	2
	Following clinical assessment, if a patients 'in-vehicle' needs can be met by FRS personnel then clinicians are recommended to withdraw from the vehicle to enable an efficient extrication	0
	FRS training in clinical care for entrapped patients should be standardised	0
	FRS and clinical personnel should be aware of the physical and observable signs of patient deterioration and if identified should make this known to the responsible clinician	0
	Within an appropriate system of training and governance, FRS personnel should be enabled to deliver in-vehicle clinical interventions that assist with extrication and mitigate avoidable patient harm	2
	Appropriate in-car interventions for the trapped patient include control of compressible haemorrhage	4
	Appropriate in-car interventions for the trapped patient include oxygen	3
	Appropriate in-car interventions for the trapped patient include decompression of tension pneumothorax	10
	Appropriate in-car interventions for the trapped patient include analgesia	3
	Appropriate in-car interventions for the trapped patient include tranexamic acid	
	Patients who require volume (fluid or blood product) resuscitation are likely to have time critical injuries and their removal from the vehicle should be prioritised. In the small number of patients who cannot be released quickly then ‘in vehicle’ fluids and /or blood products may be required	3
	The choice of blood product (where available) and IV fluids should be led by the available evidence	5
*Immobilisation* [9, 17, 28–30, 39, 45–51]	Kendrick Extrication Devices prolong extrication time and their use should be minimised	5
	Cervical collars should be loosened or removed following extrication as dictated by clinical assessment	1
	Long boards are an extrication device and are not suitable for patient carriage beyond the immediate extrication phase	1
	Pelvic slings should not be applied to patients until they have been extricated	5
	During the initial call to emergency services, patients, should be asked to self-extricate if they are able to do so and the environment is considered safe	0
	During the initial call to emergency services, bystanders should be advised NOT to assist patients with a decreased conscious level from the vehicle unless there is an immediate threat to life	1
	Call takers identifying a motor vehicle collision with suspected entrapment or patients requiring disentanglement should use an appropriately developed algorithm or call interrogation to identify the most appropriate response	3
*Patient focused extrication* [3, 4]	Communication and companionship for entrapped patients should be designated to a specific staff member who, if safe to do so and not an impediment for extrication, should join the patient in the car	0
	Where possible, patients should be referred to by name	0
	Where possible the patient should be engaged in discussion and explanation around extrication strategy and their role in this process	0
	An ‘extrication buddy’ should be assigned to explain the procedure, ensure companionship, and provide reassurance to the patient whilst entrapped	0
	Communication with the patient should be clear and use accessible lay language	0
	Where possible the ability of the public to photograph the vehicle and the patient should be minimised	3
	Attempts should be made to minimize onlooker photography and post-accident photos on social media and news channels	3
	Rescuers and their affiliated organizations should not post extrication related photos on their social media channels or websites	0
	Patients should be reassured (when true) that their co-occupants are safe (including animals)	0
	If conscious, patients should be allowed to communicate with their family members (including remotely using their phones)	0
	The potential harmful effects of social media interaction should be notified to the public / onlookers (see QR code campaign)	0
*Emergency Services Call and Triage* [34, 52–57]	On initial emergency services call attempts should be made to clarify entrapment status	0
	Consideration should be given for call back, video from scene and other modalities to enhance the fidelity of triage response	0
	Collisions identified during emergency services call as potentially requiring disentanglement should trigger a senior FRS extrication response	10
	Collisions identified at emergency services call as potentially requiring disentanglement should trigger an expert FRS extrication response	9
	Collisions identified at emergency services call as potentially requiring disentanglement should trigger an ‘enhanced^’1^ clinical care response	3
	MVC with suspected entrapment should warrant an immediate response triage category for prehospital medical services	4
	A standard multi-agency MVC trauma message should be developed to ensure the correct resources are deployed	3
	MVC with suspected entrapment should warrant an immediate response triage category for prehospital medical services	3
*Audit standards and Research* [2, 32]	Audit standards should be developed with patient and public engagement	4
	Multi-Professional (MP) datasets should be developed to enable research and audit	0
	MP datasets should include patient entrapment status	0
	MP datasets should include how badly injured and or time-critical entrapped patients are	0
	MP datasets should include different extrication approaches and their variants	0
	MP datasets should include entrapment time	0
	MP datasets should include in-car patient care and its timing	0
	MP audit standards should be developed to improve quality of patient care and extrication practice	0
Rejected statements	The rescuer goal in consideration of patient movement should be “Absolute movement minimisation and mitigation” (REJECTED)	0
	Cervical collars should be used where available on all patients as a movement minimisation tool (REJECTED)	3

*1 Enhanced care:* Enhanced care is a term used in the UK to describe a wider scope of practice above that of a non-specialist paramedic. Enhanced care may be delivered by specialist or advanced paramedics (and other clinicians) and would normally include skills such as sedation a wider choice of analgesia, enhanced decision making and other interventions

*2 Critical care:* Critical care is a term used in the UK to describe a wider scope of practice above that of enhanced care. Critical care is normally delivered by a team including specialist / advanced paramedics (or other appropriate background) and a doctor. The critical care skill set normally would include anaesthesia, surgical skills and access to blood product resuscitation

Notes on the statements:

(i) SME’s also agreed that where required to improve understanding "Where / when possible" could be added to statements

(ii) Statements / principles apply to all vehicles

(these contextual statements above were derived as individual statements from the Delphi process)

Following iterative review with individual stakeholder organisations the agreed principles for future guidance across the organisations are presented in Table 3.

**Table 3:** Principles: agreed by stakeholder organisations

Operational and clinical team members should work together to develop a bespoke patient centred extrication plan with the primary focus of minimising entrapment time
Independent of actual or suspected injuries patients should be handled gently. A focus on absolute movement minimisation is not justified
When clinicians are not available, FRSs should where necessary assess patients, deliver clinical care and make and enact extrication plans (including self-extrication)^1^
Self-extrication or minimally assisted extrication should be the standard ‘first line’ extrication for all patients who do not have contraindications, which are: -An inability to understand or follow instructions, -Injuries or baseline function that prevents standing on at least one leg, (specific injuries include: unstable pelvic fracture, impalement, bilateral leg fracture)
All patients with evidence of injury should be considered time-dependent and their entrapment time should be minimised
Incidents where a patient may require disentanglement are complex and associated with a high morbidity and mortality. A senior FRS and clinical response should attend such instances^2^
Clinical care during entrapment: -Can be delivered by FRS or clinical services^1^ -Should be limited to necessary critical interventions to expedite safe extrication^3^ -Rescuers should be aware that clinical observations may prolong entrapment time and as such should be kept to the minimum -FRS and clinical personnel should be aware of the physical and observable signs of patient deterioration and if identified should make this known to the responsible clinician
Immobilisation: -Longboards are an extrication device and should not be used beyond the extrication phase -Kedrick Extrication Devices prolong extrication time and their use should be minimised -Pelvic slings should not be applied to patients until they have been extricated -Cervical collars should only be used following assessment and should be loosened or removed following extrication
Patient focused extrication: -Build a connection with patients, explain actions, and use their name -Where appropriate, reassure patients as to the safety of their co-occupants and others involved in the incident (including animals) -Provide an ‘extrication buddy’ -Allow communication with family members or other close contacts -Rescue teams should not publish extrication related imagery to social media or other outlets -Minimise the ability of the public to view the accident, take photographs or record videos. Provide education to this effect
On initial call to Emergency Services -Attempt to clarify entrapment status -Attempt to identify patients who require disentanglement (and dispatch an appropriate priority senior^2^ response) -A standard multi-agency MVC trauma message should be developed to ensure the correct resources are deployed
Multi-professional datasets should be developed with patient and public engagement and should include entrapment status, entrapment time, injuries, extrication approach, clinical care
Agreed nomenclature for categories of patient Not injured, Minor injuries (evidence of energy transfer but no evidence of time-dependent injury), Major injury (currently stable but should be assumed to be time-dependent), Time critical injured (Time critical due to injury; use fastest route of extrication) m Time critical hazard (e.g. secondary to fire or other hazard)

*FRS* Fire and Rescue Services, *Disentanglement* requires the use of cutting tools to free patient

^1^FRS clinical care should be standardised and delivered with appropriate training and clinical governance oversight

^2^A senior or enhanced clinical and operational response should be dispatched. This may include enhanced / critical care and will benefit from further consideration

^3^In-car interventions may include the administration of tranexamic acid, analgesia and oxygen. Interventions may include the management of compressible haemorrhage and decompression of suspected tension pneumothorax. Patients who require volume (fluid or blood product) resuscitation are likely to have time critical injuries and their removal from the vehicle should be prioritised. In the small number of patients who cannot be released quickly then ‘in vehicle’ fluids and /or blood products may be required

## Discussion

This Delphi study achieved consensus on 91 statements in an area of previously limited multidisciplinary, evidence-based guidance. These statements will provide a vital foundation for the development of multidisciplinary consensus guidance and best practice standards for the extrication of patients trapped in motor vehicles following a collision. These statements have been effectively translated into agreed multidisciplinary principals.

A key principle agreed by the SMEs identifies that operational and clinical team members should work together to develop a bespoke patient centred extrication plan with the primary focus of minimising entrapment time. The SMEs rejected the historical focus on absolute movement minimisation and instead recommended gentle patient handling for all patients independent of actual or suspected injuries. The SMEs encouraged FRS team members to take an active role in assessing patients, delivering clinical care and enacting extrication plans (including self-extrication). Inclusion and exclusion criteria for self-extrication or minimally assisted extrication were identified and agreed.

SMEs reached consensus in Round 1 in all the statements in the domain areas: ‘Patient focused extrication’ and ‘Audit standards and Research’. Consensus was also reached following Round 1 across all statements in the theme areas: ‘Terminology’, ‘Extrication Goals and Approach’ and ‘Patients requiring Disentanglement’. Consensus was not achieved for some of the remaining domain areas with the most contentious being the risk stratification of patients for potential cervical spinal injury, which patients should have a collar applied, and which professional groups should be providing “in vehicle” clinical care for those that remained trapped. The subject of immobilisation, patient handling and the use of cervical collars has received much attention in the literature; with increasing acknowledgement of the incomplete evidence base for historic approaches and the support of pragmatic alternative approaches [9, 17, 28–30, 39, 45–51]*.* These themes are explored in more detail in the SME briefing document included in the Additional file.

In general terms the SMEs were quicker or more likely to reach consensus in areas of practice where there was little evidence available or there was no current guidance e.g. patient focused extrication (Additional file 1). When there was more evidence available or in areas where there is current (often contradictory) guidance, the SME’s less frequently achieved consensus [7–9, 27]. This tension was displayed by more SME’s choosing to ‘opt out’ of the evidence rich statements, but the divergence in opinion of those that did participate remained consistent through the 3 rounds.

Consensus was harder to achieve in areas where professional roles and patient ‘ownership’ needed to be considered. Historically medical care has been delivered by clinicians with a health care background with rescue workers only offering minimal clinical assessment and interventions [4, 58, 59]. Recently in clinical and operational practice these boundaries have become more fluid with rescue services offering more clinical development to their personnel [59]. The statements in this Delphi considered the role of rescue services in delivering this care which was met with strong and diverse opinions. Through the rounds of the Delphi, the purpose of the statements was clarified, this along with clearer alternative statements led to consensus being achieved.

The utilisation of the CREDES Delphi standards for this study ensured that it was conducted and reported to an appropriate standard [10]. The SMEs demonstrated a high participation rate in the process with 82% of the original SMEs completing all three rounds. This Delphi was unusual both in the high number of statements presented to the SMEs and the high level of concordance between the SMEs leading to many statements reaching consensus. We took several steps to ensure that our SME selection was robust, unbiased and with minimal sampling bias, but our SMEs may not be truly representative of wider expertise in this subject area, and this may affect the external validity of our results. The CREDES standards support the piloting of questions with SMEs, the influence this may have on our results is unknown. All SMEs were drawn from a UK rescue service or prehospital clinical background and therefore these results may not be valid in countries with significant differences in availability or structure of rescue or clinical provision. It may be appropriate to reproduce some elements of this Delphi for settings which are notable different e.g. lower and middle income countries or military environments.

Following this Delphi, further work will be needed to support the translation of the principles into practice. Some domains from the Delphi will require further clarification; the SMEs identified the following areas for further consensus work: FRS clinical training (87.8%), collars and immobilisation (75.5%), EMS call handling and dispatch (73.5%), and self-extrication (63.3%).

The principles agreed by stakeholder organisations will offer a basis on which future discipline specific guidance will be based. The variability in format, language, scope and approach between clinical and rescue guidance prevents the production of a single cohesive guideline that would meet the needs of all the stakeholder organisations. Further translational work will ensure that the principles developed here are embedded in Joint Royal Colleges Ambulance Liaison Committee (JRCALC) guidance to guide paramedics, FPHC guidance to guide advanced prehospital emergency medicine practitioners and National Operational Guidance (NOG) to guide rescue services.

The principles established in this Delphi benefit from having minimal financial costs associated with bringing them into practice. We envisage the main barrier to adoption of new guidance will be overcoming the institutional and individual inertia established through 50 years of movement minimisation based clinical and operational practice—the challenges of unlearning cannot be underestimated [60]. The adoption of a formal evidence-to-practice process such as the Knowledge to Action (KTA) framework will help guide which steps will be most effective in the next challenging phase of this work [61–63].

The stakeholders represented in this Delphi will need to continue to work together to refine these principles for guidance and continue to revise their guidance based on the feedback of early adopters and audit outcomes from longitudinal data collection.

## Conclusion

This study has demonstrated consensus across a large panel of multidisciplinary SMEs on many key areas of extrication and related practice that will provide a key foundation in the development of evidence-based guidance for this subject area.

## Supplementary Information

**Additional file 1.** Subject Matter Expert (SME), Briefing and evidence summary.

## Data Availability

The datasets used and/or analysed during the current study are available from the corresponding author on reasonable request.
