# Supplementary material for: A Delphi study of rescue and clinical subject matter experts on the extrication of patients following a motor vehicle collision
Source: Scand J Trauma Resusc Emerg Med. 2022 Jun 20;30:41. doi: 10.1186/s13049-022-01029-x (PMC9208189; doi:10.1186/s13049-022-01029-x)
Supplement: Supplementary file 1 — Additional file 1. Subject Matter Expert (SME), Briefing and evidence summary. [file 13049_2022_1029_MOESM1_ESM.pdf]

Subject Matter Expert (SME), Briefing and evidence summary

Version 1.1.
December 2021

An evidence-based approach to extrication: a Delphi Study

Many thanks for your interest in this study. This study will use Delphi techniques* to find consensus on an “evidence-based approach to extrication”.

This study output is sponsored by the Road Safety Trust and is delivered in collaboration with the National Fire Chiefs Council (NFCC), the United Kingdom Rescue Organisation (UKRO), the National HEMS Research & Audit Forum (NHRAF), the College of Paramedics (CoP), the Pre-Hospital Trainee Operated Research Network (PHOTON) and the Faculty of Prehospital Care (FPHC).

This study has received ethical approval from the University of Plymouth, Faculty Research Ethics and Integrity Committee (ref. 19-20-1313).

This document:

- Part A: Explains how the study will work
- Part B: Provides an overview of the evidence base for and the statements you will be asked to consider in each round of the Delphi.

*The Delphi survey is a group facilitation technique, which is an iterative multistage process, designed to transform opinion into group consensu**s.**

PART A: THE DELPHI PROCESS

What is a Delphi survey:
The Delphi survey is a group facilitation technique, which is an iterative multistage process, designed to transform opinion into group consensus. It is commonly used when other forms of trials / evidence gathering are unsuitable. The Delphi technique is also used in the consensus building phase where evidence from multiple sources must be combined with expert opinion to deliver practical guidance.

Why have I been asked to be involved:
You have been identified as a Subject Matter Expert (SME) in the field of Extrication. You have been invited to participate as you have been nominated by one of the partnership organisations or by another SME.

One of the features of the Delphi is that participants remain anonymous from each other - this encourages the free expression of expert views and minimises bias in the consensus process.

What happens next:
This introductory document outlines the process for participation. The consent and submission processes are completed "online"; all communication will be via the email address that you have supplied.

This Delphi study will consist of up to three rounds.

Round 1: In this round you will be asked to consider the statements in the Part B of this document. For each statement you can Agree, Disagree, Neither Agree nor Disagree or Opt out. Please only use the "Opt out" function if you feel that you don't have the expertise to comment on that specific area.

- Statements which achieve 70% Agree or Disagree will be considered to have reached consensus for agreement or rejection.
- At the end of each section there is an opportunity to suggest modifications to existing questions or to submit additional questions for the consensus process. All these submissions will be considered by the Steering Group and added to the Round 2 if considered to add utility.

Dates for round 1: 3-14 January 2022.

Subsequent rounds: Statements that fail to reach consensus for agreement or rejection along with selected additional or clarifying statements will be carried through to subsequent rounds.

Planned dates:
Round 2: 24 Jan - 4 Feb 2022
Round 3: 14 - 25 Feb 2022

Additional information:

This Delphi study has a place in a larger package of work with the overarching aim for developing evidence based guidance for the extrication of casualties trapped following a Motor Vehicle Collision (MVC). Data from the contributory studies has been used in preparing the evidence reviews.

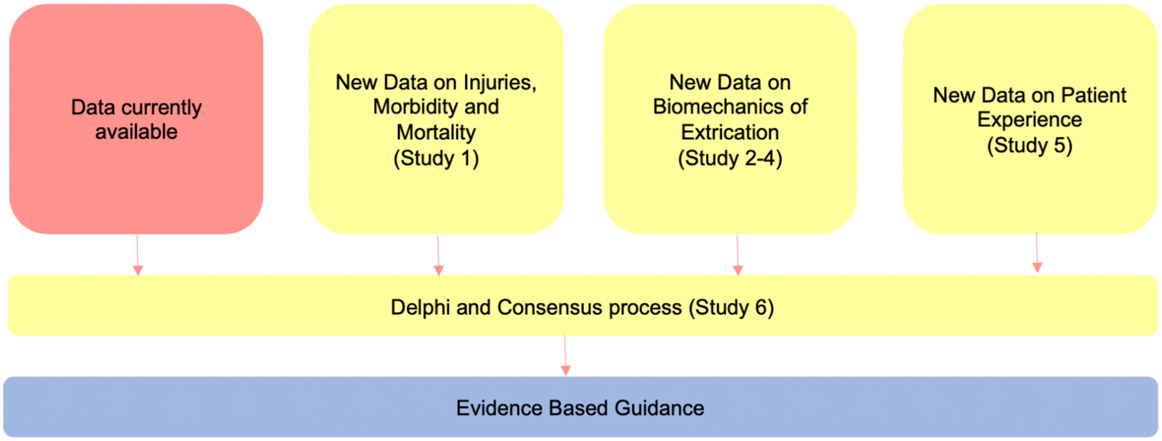

Contacting the study team:
Tim Nutbeam can be contacted on timnutbeam@nhs.net or on 07779 231 813

PART B: Evidence reviews and statements for consideration

This document is split into the following sections:

Terminology
Extrication goals and approach
Self-extrication
Disentanglement
Clinical care
Immobilisation
Casualty focused extrication and post extrication care
Triage and 999 call
Audit standards and research
References

Each section consists of a brief evidence review (full references are provided*) and the statements for consideration in Round 1.

*At previous stages of this project, relevant questions have been considered and the literature summarised. To maximise accessibility not all original references have been included but are available within the referenced papers or on request to the study team.

Section 1: Terminology

This section relates to the terminology utilised by the wide range of professionals attending a motor vehicle collision (MVC) and considering whether standardisation of terminology would be preferable and should be encouraged.

A review of national and international extrication related literature from a clinical and Fire and Rescue Services (FRS) perspective identified a range of language, nomenclature, terminology and approaches [1–7] (selected references).

Standardisation of terminology in healthcare settings has been found to be beneficial in the context of patient safety and is widely encouraged by WHO, NICE and others [8].

Organisations such as JESIP encourage pan-professional language, communication and guidance to improve interoperability [9].

Terminology statements

- A multi-professional, standardised terminology should be developed and adopted to describe different extrication approaches and their variants
- A multi-professional, standardised terminology should be adopted to describe risks and hazards at a scene of an entrapped casualty
- A multi-professional, standardised terminology should be adopted to described how unwell and or time-critical entrapped casualties are
- A multi-professional, standardised terminology should be developed and adopted to describe the entrapment status of casualties (e.g. medically trapped, physically trapped)

Section 2: Extrication goals and approach

Extrication, injuries and mortality

Motor vehicle collisions (MVCs) are a common cause of injury and death [10]. Following a MVC, casualties that remain trapped in their vehicles have more severe injuries and are more likely to die [11].

Table 1: Taken from Nutbeam et al. Scandinavian Journal of Trauma, Resuscitation and Emergency Medicine (2021) 29:17 https://doi.org/10.1186/s13049-020-00818-6

Table 1 Demographics and Mortality by Trapped Status

	Trapped		Not trapped		Sig /*p* value
Number of patients (%)	6983	(11.0%)	56,642	(89.0%)	–
Male n (%)	4374	(62.6%)	42,656	(75.3%)	–
Mean Age years (STD DEV)	44.2	(21.3)	43.4	(21.3)	0.003
Median ISS (IQR)	18	(10–29)	13	(9–22)	< 0.001
Systolic Blood Pressure mmHg (STD DEV)	129	(31)	133	(27)	< 0.001
Respiratory Rate (STD DEV)	21	(7.9)	20	(6.8)	< 0.001
Oxygen Saturations % (STD DEV)	94.8%	(10.5)	96.3%	(7.5)	< 0.001
Median GCS (IQR)	15	(14–15)	15	(13–15)	< 0.001
Crude 90 day mortality n (%)	624	(8.9%)	2804	(5.0%)	< 0.001

Table 2: Taken from Nutbeam et al. Scandinavian Journal of Trauma, Resuscitation and Emergency Medicine (2021) 29:17 https://doi.org/10.1186/s13049-020-00818-6

Table 2 Time-critical and Spinal Injuries by Trapped Status

	Trapped		% of all Extrications[a]	Not trapped		Sig / *p* value:
Pelvic ring with blood loss > 20% n (%)	69	(1.0%)	0.16	370	(0.7%)	0.001
Blood loss > 20% n (%)	244	(3.5%)	0.56	1057	(1.9%)	< 0.001
Tension Pneumothorax n (%)	105	(1.5%)	0.24	472	(0.8%)	< 0.001
Multiple Spinal Fractures n (%)	942	(13.5%)	2.16	5003	(8.8%)	< 0.001
Spine Dens: Fracture n (%)	146	(2.1%)	0.33	586	(1.0%)	< 0.001
Spine: Compression Fracture n (%)	118	(1.7%)	0.27	1006	(1.8%)	0.606
Spine: Unstable Fracture n (%)	635	(9.1%)	1.46	3583	(6.3%)	< 0.001
Spine: Cord Injury n (%)	464	(6.6%)	0.71	2687	(4.7%)	< 0.001

[a]Percentage of all extrications performed during matched time period from FRS data

Extrication is the process of removing casualties with known or potential injuries from their vehicles [11,12]. Rescue services have developed a wide range of techniques to enable access to casualties and extricate them from their vehicles [12].

Movement mitigation

The historical approach to extrication is based on movement minimisation and mitigation, primarily to avoid exacerbating a primary spinal injury [13].

The role of small movements in this context is unknown and a challenge to accurately quantify [14]. Large or forceful movements are considered higher risk than smaller movements [15].

Many 'traditional' extrication techniques have developed with a primary focus of movement minimisation because of concerns related to the potential for excessive movement either causing or contributing to secondary spinal injury [13]. Movement minimisation during extrication comes at the expense of time, with extrications on average taking in excess of 30 minutes [16,17].

Some rescue service teaching recommends that a majority of casualties receive a traditional extrication method, as it is understood that these result in less spinal movement than other techniques [12,13]. Recently these principles have been challenged; with a number of biomechanical studies demonstrating that self-extrication may cause less movement than more traditional extrication techniques [18–20].

Movement by extrication type

Work to support this project has delivered powered studies comparing cervical and lumbar spine movement across a range of extrication types [21,22]. The findings for anterior posterior (AP) movement at the cervical spine are shown below. The trend in results demonstrated with AP movement is consistent across all movement directions considered. The groups considered were:

Figure: Anterior Posterior Cervical Spine Movement and Extrication Type

(*Error bars indicate 95% Confidence Intervals)

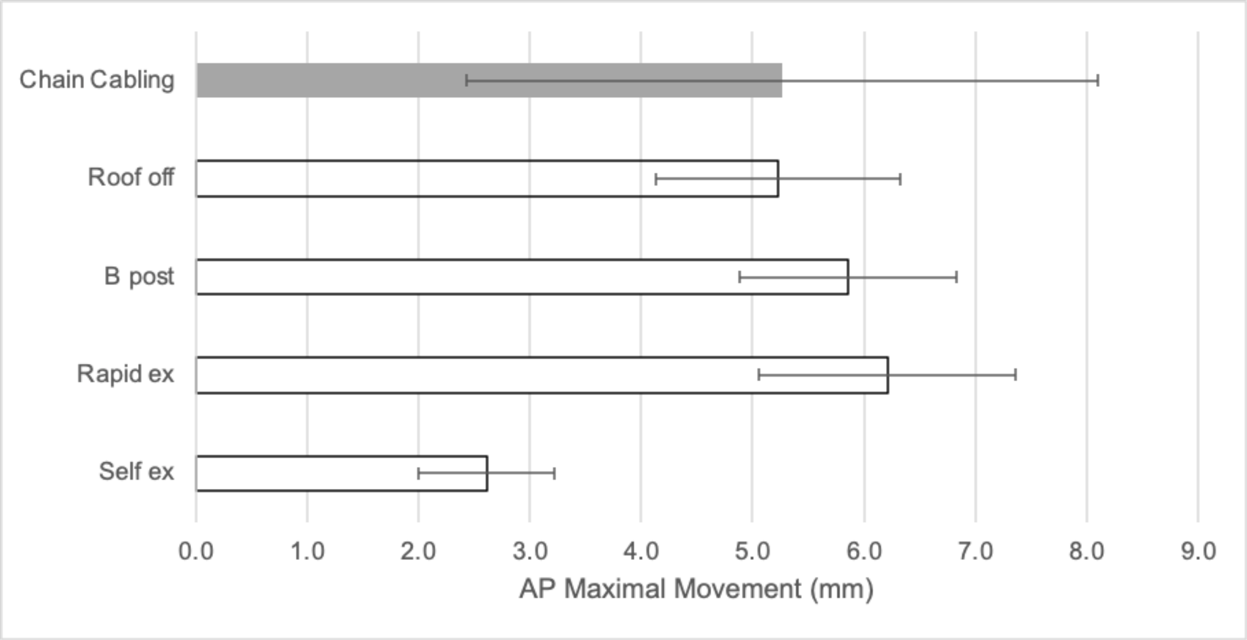

Figure: Mean excursion and 95% confidence intervals for anterior-posterior movement at the cervical spine

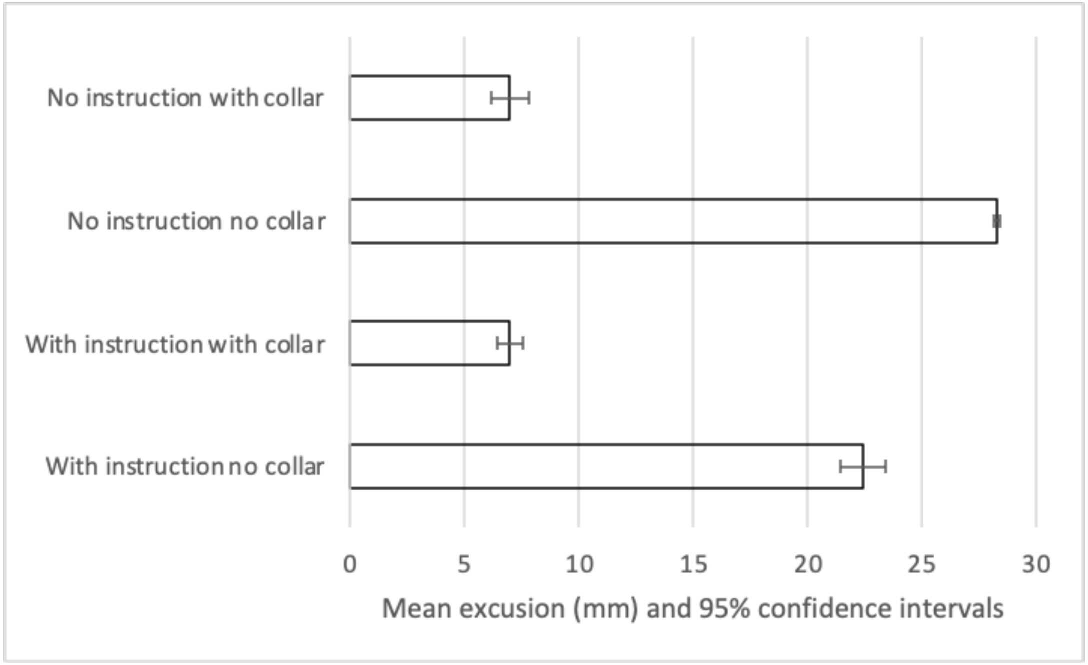

Spinal injuries and other time critical injuries

(adapted from Nutbeam et al. Scandinavian Journal of Trauma, Resuscitation and Emergency Medicine (2021) 29:17)

Spinal injuries are infrequent in studies which consider their rate in trapped patients [11]. In this particular study trapped patients with a spinal cord injury representing just 0.71% (or one in 141) of all extrications performed. For the very small proportion of patients whom 'traditional' extrication techniques are targeted towards there is a very large number of patients with no or minor injuries whom as a result of application of movement minimisation techniques consume significant resources. In addition, there is a large number of severely injured patients who have non-spinal or spinal and additional injuries in who extrication approaches are not optimised for.

Table 2: Taken from Nutbeam et al. Scandinavian Journal of Trauma, Resuscitation and Emergency Medicine (2021) 29:17

https://doi.org/10.1186/s13049-020-00818-6

Table 2 Time-critical and Spinal Injuries by Trapped Status

	Trapped		% of all Extrications[a]	Not trapped		Sig / *p* value:
Pelvic ring with blood loss > 20% n (%)	69	(1.0%)	0.16	370	(0.7%)	0.001
Blood loss > 20% n (%)	244	(3.5%)	0.56	1057	(1.9%)	< 0.001
Tension Pneumothorax n (%)	105	(1.5%)	0.24	472	(0.8%)	< 0.001
Multiple Spinal Fractures n (%)	942	(13.5%)	2.16	5003	(8.8%)	< 0.001
Spine Dens: Fracture n (%)	146	(2.1%)	0.33	586	(1.0%)	< 0.001
Spine: Compression Fracture n (%)	118	(1.7%)	0.27	1006	(1.8%)	0.606
Spine: Unstable Fracture n (%)	635	(9.1%)	1.46	3583	(6.3%)	< 0.001
Spine: Cord Injury n (%)	464	(6.6%)	0.71	2687	(4.7%)	< 0.001

[a]Percentage of all extrications performed during matched time period from FRS data

[illegible]

Do patients with spinal injuries have other injuries which may dictate extrication needs?

(adapted from Nutbeam et al. Scandinavian Journal of Trauma, Resuscitation and Emergency Medicine (2021) 29:17)

In the context of prevention of secondary spinal injury, those patients who may benefit from movement minimisation are those who have a spinal cord injury and do not have other time-critical injuries that may take precedence when planning an extrication. This is a rare patient group; just 232 patients over the 6 years that this study covers, or 0.5% of the 43,633 total extrications that occurred. As isolated cord injury represents a small proportion of those who are trapped, extrication principles should be reconsidered with a wider appreciation of the mortality and morbidity associated with other common injuries and injury patterns. Within our data, for example, a trapped patient is five times more likely to have a chest AIS of 3+ than a spine AIS of the same severity (Table 3).

Table 3: Taken from Nutbeam et al. Scandinavian Journal of Trauma, Resuscitation and Emergency Medicine (2021) 29:17

https://doi.org/10.1186/s13049-020-00818-6

Table 3 Injury site by Trapped Status

Injury site[a]	Trapped		% of all Extrications[b]	Not trapped		Sig / *p* value:
Head AIS > = 3, n (%)	1742	(25.0%)	3.99	13,060	(23.1%)	< 0.001
Face AIS > = 3, n (%)	48	(0.7%)	0.11	307	(0.5%)	0.124
Chest AIS > = 3, n (%)	3699	(53.0%)	8.48	19,624	(34.7%)	< 0.001
Abdo AIS > = 3, n (%)	858	(12.3%)	1.97	4299	(7.6%)	< 0.001
Pelvis AIS > = 3, n (%)	738	(10.6%)	1.69	3487	(6.2%)	< 0.001
Spine AIS > = 3, n (%)	795	(11.4%)	1.82	4208	(7.4%)	< 0.001
Limb AIS > = 3, n (%)	2275	(32.6%)	5.21	16,668	(29.4%)	< 0.001

[a]Injuries are not mutually exclusive; patients may have more than one qualifying injury
[b]Percentage of all extrications performed during matched time period from FRS data that had these injuries

Age and (self) extrication

Patients over the age of 80 are more likely to die when trapped following an MVC [23].

Figure : Age and adjusted odds of death [23]*

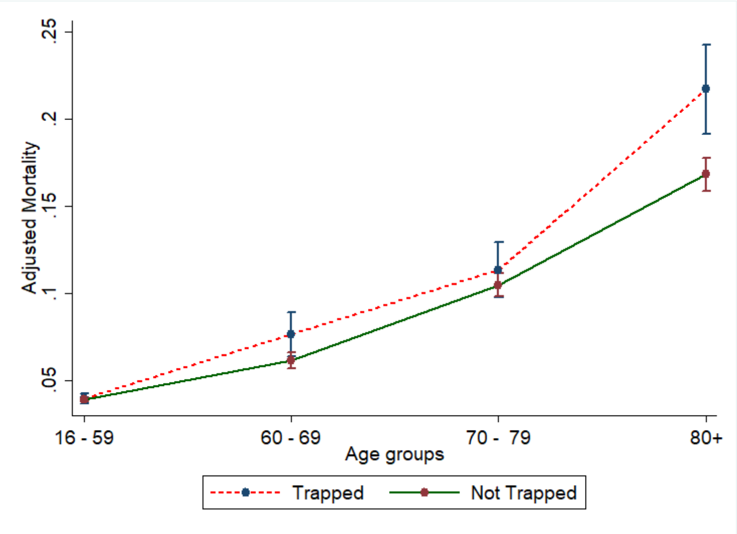

*Logistic regression was used to develop a model with the following known confounders: gender, ISS, GCS, Charlson comorbidity index and entrapment status as exposure variables, considering the odds of death by age group, and examining any interaction between age and trapped status with mortality.

Casualties have similar potential for self-extrication regardless of age:

[illegible]

Physiological and injury considerations for potential for self-extrication by age

		Age group				
**Parameter**	Total	16 - 59	60 - 69	70 - 79	80+	Significance (p)
Systolic BP <90	418 (5.4%)	301 (5.3%)	48 (6.0%)	39 (5.2%)	30 (5.5%)	0.908
GCS 12 or less	1183 (15.3%)	1006 (17.8%)	68 (8.4%)	57 (7.5%)	52 (9.5%)	< 0.0001
Spine AIS3+	844 (10.9%)	577 (10.2%)	96 (11.9%)	109 (14.4%)	62 (11.3%)	< 0.0001
Pelvic AIS 3+	895 (11.5%)	686 (12.2%)	82 (10.2%)	60 (7.9%)	67 (12.2%)	< 0.0001
Limb AIS 3+	2522 (32.5%)	2028 (35.9%)	232 (28.7%)	164 (21.7%)	98 (17.9%)	< 0.0001
None of the above	3208 (41.4%)	2264 (40.1%)	343 (42.5%)	357 (47.2%)	244 (44.4%)	0.079

Statements: Extrication goals and approach

- The historical focus on absolute movement minimisation is no longer justified given information on rarity of spinal injury and frequency of other time critical injuries
- The rescuer goal in consideration of casualty movement should be “Gentle casualty handling’
- The rescuer goal in consideration of casualty movement should be “Absolute movement minimisation and mitigation’
- Minimising entrapment time should be a multi-professional goal for all entrapped casualties
- Self-extrication or minimally assisted extrication should be the standard ‘first line’ extrication for entrapped casualties who are conscious and likely to be able to stand with assistance
- Extrication routes (other than self-extrication) appear to be biomechanically similar, so it is reasonable to choose the quickest deliverable route given the specific circumstances of the incident
- Unconscious casualties have high risk of significant injuries and should have an expedited extrication undertaken using ‘gentle casualty handling’ techniques
- Casualties with acute neurological deficit (e.g., pins and needles in arms) require expedited extrication to allow for thorough assessment and management of injuries
- FRS should begin the process of extrication regardless of the presence of clinicians on scene
- FRS should take overall responsibility for coordination of the extrication
- Extrication goals and approach should be similar regardless of the sex or gender of a casualty
- Extrication goals and approach should be similar regardless of the age of a casualty

Section 3: Self-extrication

A number of studies (in healthy volunteers) have found self-extrication to be biomechanically superior (in terms of cervical and lumbar spine movement) to other extrication methods [18,19,21]. No studies have found that self-extrication is biomechanically inferior to other extrication techniques.

Figure: Anterior Posterior Cervical Spine Movement and Extrication Type [21]

(*Error bars indicate 95% Confidence Intervals)

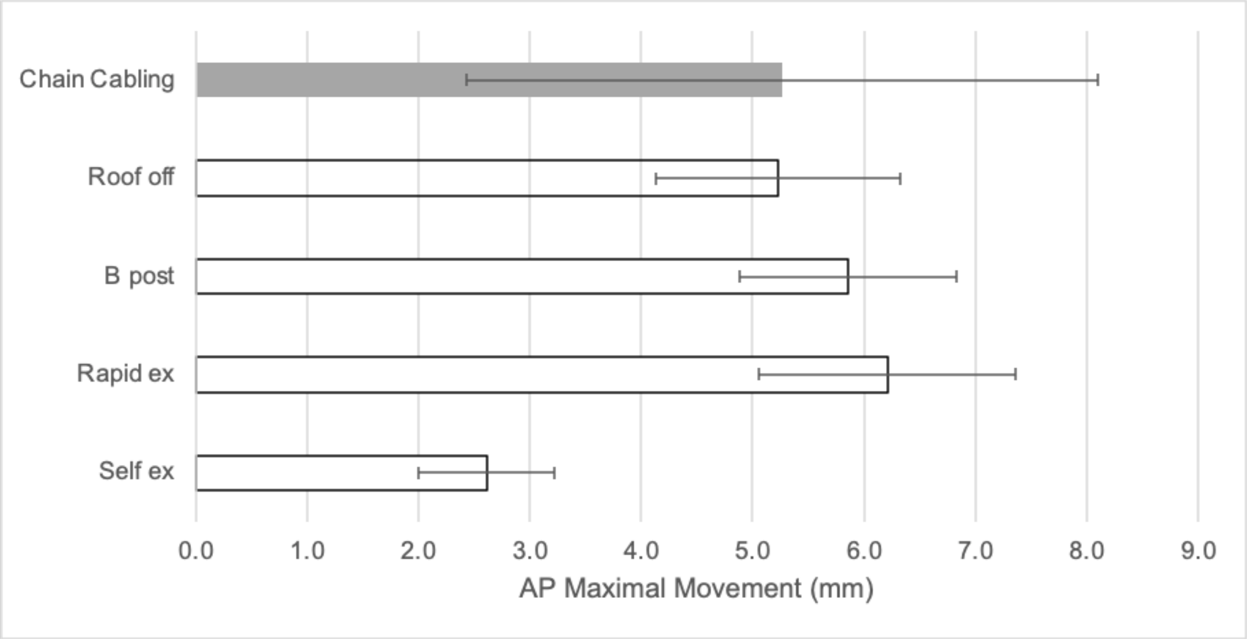

Self-extrication is quicker than other extrication types [21].

The cervical spine movement associated with self-extrication is reduced by the application of a collar [22]. Collars may not be necessary for patients with and without neck injuries who may be suitable for self-extrication [14,24,25].

Figure: Mean excursion and 95% confidence intervals for anterior-posterior movement at the cervical spine[22]

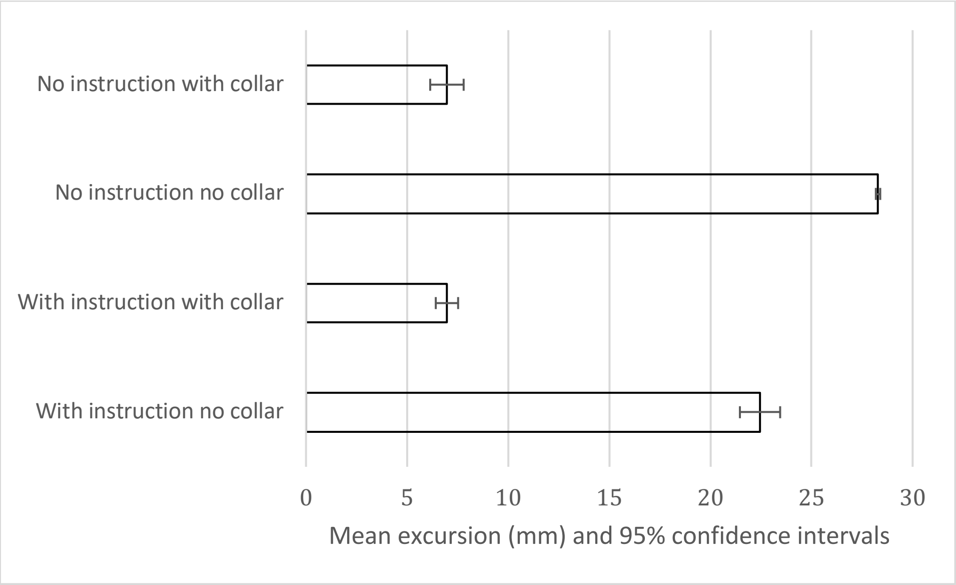

Casualties have similar potential for self-extrication regardless of age:

Physiological and injury considerations for potential for self-extrication by age

		Age group				
**Parameter**	Total	16 - 59	60 - 69	70 - 79	80+	Significance (p)
Systolic BP <90	418 (5.4%)	301 (5.3%)	48 (6.0%)	39 (5.2%)	30 (5.5%)	0.908
GCS 12 or less	1183 (15.3%)	1006 (17.8%)	68 (8.4%)	57 (7.5%)	52 (9.5%)	< 0.0001
Spine AIS3+	844 (10.9%)	577 (10.2%)	96 (11.9%)	109 (14.4%)	62 (11.3%)	< 0.0001
Pelvic AIS 3+	895 (11.5%)	686 (12.2%)	82 (10.2%)	60 (7.9%)	67 (12.2%)	< 0.0001
Limb AIS 3+	2522 (32.5%)	2028 (35.9%)	232 (28.7%)	164 (21.7%)	98 (17.9%)	< 0.0001
None of the above	3208 (41.4%)	2264 (40.1%)	343 (42.5%)	357 (47.2%)	244 (44.4%)	0.079

Case reports highlight that casualties even with significant injuries, including spinal injuries have the potential for self-extrication [26].

The parameters for which patients should be offered self-extrication have not been defined or validated [23].

Statements: Self-extrication

- All casualties should be assessed to see if they are suitable for self-extrication as the primary method of extrication
- Casualties with neck and / or spinal pain should be considered for self-extrication
- Casualties with evidence of neurological deficit (e.g., pins and needles in arms) should be considered for self-extrication
- Casualties with lower limb injuries should be considered for assisted self-extrication
- Casualties with suspected pelvic injuries should be considered for assisted self-extrication
- Casualties regardless of their injuries should be assessed for suitability for (assisted) self-extrication
- (Narrative question) Injuries or findings which would preclude a casualty from self-extrication include …………

Section 4: Disentanglement

True physical / anatomical entrapment requiring disentanglement is infrequent (<10% of extrications)[27].

Intrusion into the vehicle is associated with physical / anatomical entrapment and major trauma or injury [28–31].

Casualties have a longer entrapment time and scene time when disentanglement is required [3,6,12,16,27,30,32,33].

Statements: Disentanglement

- Casualties who are physically entrapped as a result of intrusion have a high likelihood of significant injuries and as such should be considered time critical
- Disentanglement should be followed by the quickest appropriate extrication type
- Disentanglement should be followed by the quickest appropriate extrication type including self-extrication
- Collisions where casualties require disentanglement should trigger a senior FRS extrication response
- Collisions where casualties require disentanglement should trigger an expert FRS extrication response
- Collisions where casualties require disentanglement should trigger a ‘enhanced’ clinical care response
- Collisions where casualties require disentanglement should trigger a ‘critical-care’ clinical care response
- Post-extrication casualties who have required disentanglement should be transferred preferentially to a major trauma centre/ form part of the major trauma triage tool

Section 5: Clinical care

Studies concerning in-car clinical care are limited to expert opinion, manikin studies and case reports [1,3,4,34–38]. Many of these studies are some years old and do not reflect current clinical practice.

Statements: Clinical care

- Entrapped casualties with evidence of energy transfer (injury) should be considered to have time-dependent injuries and entrapment time should be minimised
- Clinical care should be kept to the minimum to expedite extrication
- Repeated clinical observations of an entrapped casualty slow extrication time and are unlikely to change management and as such should be minimised or avoided altogether
- Clinical procedures such as intubation and thoracostomy should ideally be delayed until a casualty has been extricated
- Appropriate in-car interventions for the trapped casualty include tranexamic acid
- Appropriate in-car interventions for the trapped casualty include analgesia
- Appropriate in-car interventions for the trapped casualty include oxygen
- Appropriate in-car interventions for the trapped casualty include IV fluids
- Appropriate in-car interventions for the trapped casualty include blood product resuscitation
- Appropriate in-car interventions for the trapped casualty include control of compressible haemorrhage
- Appropriate in-car interventions for the trapped casualty include decompression of tension pneumothorax
- In the absence of appropriately trained clinicians FRS should be enabled to deliver in-car interventions
- Primary clinical care in-car should be provided by an appropriately qualified member of the FRS unless immediate additional clinical support is required

- Post-extrication casualties who were entrapped should be transferred preferentially to a major trauma centre/ form part of the major trauma triage tool

Section 6: Immobilisation during extrication

Spinal immobilisation is a subject of some controversy with a divergence in international prehospital practice [14,24,25,39–45].

Historical absolute movement mitigation approach to spinal immobilisation has been de-emphasised within clinical practice and guidance.

The cervical spine movement associated with self-extrication is reduced by the application of a collar [22]. Collars may not be necessary for patients with and without neck injuries who may be suitable for self-extrication [14,24,25].

Figure: Mean excursion and 95% confidence intervals for anterior-posterior movement at the cervical spine[22]

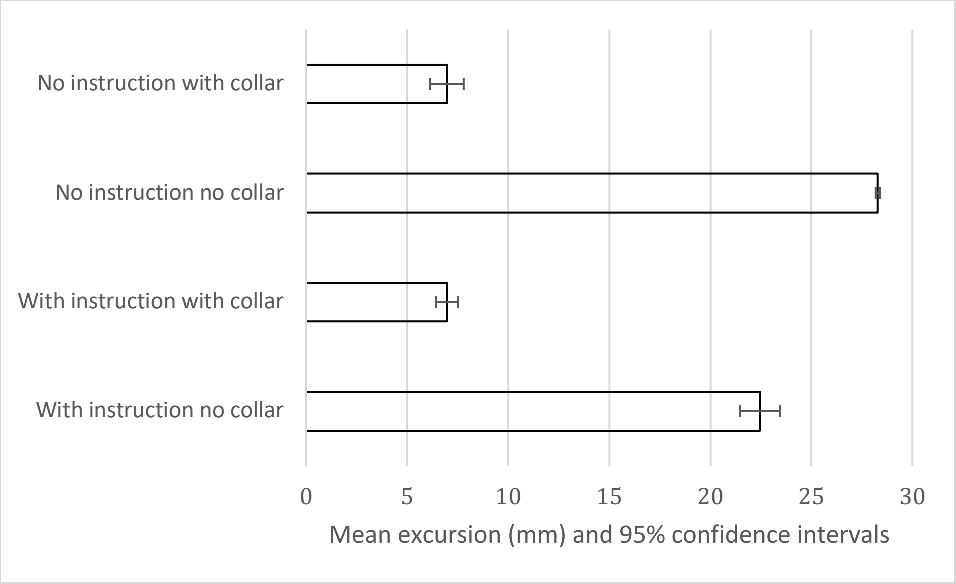

Casualties have similar potential for self-extrication regardless of age [23].

Statements: Immobilisation during extrication

- Cervical collars should be used where available on all casualties as a movement minimisation tool
- Cervical collars should be used where available on casualties with neck pain as a movement minimisation tool
- Cervical collars should be used where available on casualties with neurological deficit (e.g. pins and needles in arms) as a movement minimisation tool
- Cervical collars should be used where available on casualties with decreased conscious level as a movement minimisation tool
- Following application of a risk assessment tool (e.g. NEXUS / Canadian C-Spine rule) then cervical collars should be used when available and indicated by the tool
- Cervical collars should be removed following extrication
- Long boards are an extrication device and are not suitable for casualty carriage beyond the immediate extrication phase
- Given the low frequency of open book type pelvic fractures and the additional added time to extrication the use of pelvic slings should be minimised
- Kendrick Extrication Devices prolong extrication time and their use should be minimised

Section 7: Casualty-focused extrication and post-extrication care

There is little evidence of casualty engagement and feedback within current guidance and manuals [12,46].

The evidence for this section comes from an as yet unpublished thematic analysis of interviews from casualties who had been extricated following a motor vehicle collision.

Selected categories, codes and quotes are included in the table below to inform the Delphi panel of key themes in this area.

<u>Reporting of accident details on social media contributes to distress for the casualty and their family.</u>

"But I've not been strong enough to see those photos yet. I've not seen them." (6)

" I had messages coming from all kinds of people that I hadn't spoken to in ages…. It was a bit overwhelming" (8)

"I was shocked by the pictures" (5)

<u>Pain experience does not match patient expectation and has high inter and intra casualty variability</u>

"It started hurting after a little while but at that point, nothing hurt" (3)

"If I'd known at that particular point that, y'know, how serious it was, [laughs] I might've (felt?) a bit different or even more panicky" (9)

"the only bit I can remember being super painful." (8)

"you know when you're in pain you know and (something's?) hurt" (7)

" taking me out of the vehicle was quite quick, was painless." (9)

<u>Casualties frequently experienced a "freeze" phase after collision characterised by lack of pain and unawareness of injury.</u>

"I thought oh my god, they're gonna think I'm dead. Cos I'm in this bubble and I can't get out" (6)

"I could hear my Mum saying, [Name], are you OK? [Name], are you OK? And I couldn't move." (6)

" I could hear this little voice going [in a small voice] help me, please, help me. And I was like (.) where's that voice coming from? And it, oh god, that's me." (3)

Casualties frequently experienced distortion in memories

"it was like sitting with a jigsaw puzzle. And some bugger had about five or six pieces out of this. Y'know, I've got all these pieces here, because I couldn't put it together." (2)

"not a blur completely, but (.) it all just sort of I don't know what happened in what order" (3)

"So I think it is just my head not letting me deal with it:". (6)

A "rescue" companion contributes to a positive emotional state in the casualty

"she was gonna get out of the car, and I said to her, y'know, do you think you could stay with me? And from that moment on she didn't leave my side" (5)

"And I just felt really safe" (3)

"she was brilliant just talking me through it, just to take my mind off it. And just assuring me that my dad and everybody was OK" (8)

"reassuring for you, then, to have somebody with you - Very. Yes. Yeah" (5)

"And they were just talking about normal stuff, about my day and what I was having for lunch, and stuff. And you know, it just took my mind off it and made it a bit easier" (8)

"I was going, I can't breathe, I can't breathe, I can't breathe. And she was just saying, just keep calm; you're doing well; you're fine; you're doing all right (6)

Poor communication skills by rescuers led to a loss of agency by the casualty and a negative extrication experience

"I was like amazed how (.) they didn't talk to me once, even the fire service or the police or (.) the ambulance service.." (7)

"And I kept saying, can you please get my phone…. They're like yeah, the police are doing that, it's all sorted; police are doing it" (family communication didn't occur) "And that has [audible inhalation] that has traumatised him." (3)

"I knew I had to get out the car, but I think it wasn't discussed with me how it was going to occur." (9)

"they should've just like listened to me instead of like making their own assumptions." (7)

"they didn't tell me that they were cutting the car open. So I started panicking even more." (7)

Explanations, warmth and human communication with casualty and family contributed to a positive extrication experience

"Made sure I was nice and warm. (.) I think they were brilliant."

"you're in a car, just after an accident, you still need that reassurance that nothing else is gonna happen." (7)

"especially when they said they'd take care of my dog, which was my top priority."

"They told me whenever they were going to do anything what they were going to do, why they were gonna do it"

"once I had the information to put that at rest, I felt, y'know, that that was alleviated"

"And he’d got a visor on. Because I was like lying on the floor. It would’ve been nice to see somebody’s face." (2)

Statements: Casualty focused extrication and post extrication care

- Where possible casualties should be referred to by name
- Where possible the casualty should be engaged in discussion and explanation around extrication strategy and their role in this process
- Communication and companionship for entrapped casualties should be designated to a specific staff member. If safe to do so and not an impediment for extrication they should join the casualty in the car.
- Communication with the casualty should be clear and use accessible lay language
- An ‘extrication buddy’ should be assigned to explain the procedure, ensure companionship and provide reassurance to the casualty whilst entrapped
- Where possible the ability of the public to photograph the vehicle and the casualty should be minimised
- Attempts should be made to minimise onlooker photographer and post-accident photos on social media and news channels
- Rescuers and their affiliated organisations should not post post-extrication photos on their social media channels or websites
- Casualties should be reassured (when true) that their co-occupants are safe (including animals)
- If conscious, casualties should be allowed to communicate with their family members
- The potential harmful effects of social media interaction should be notified to the public / onlookers (see QR code campaign)

Section 8: Triage and 999 call

Early dispatch of appropriate resources to MVCs is associated with improved system performance and patient outcome [29,47–50].

Triage systems for MVC with entrapment are based around closing speed (high v low speed), low risk features (such as ‘fender bender’) and the presence of intrusion or entrapment [29,49–52].

Statements: Triage and 999 call

- On initial 999 call attempts should be made to clarify entrapment status
- On initial 999 attempts should be made to identify casualties that will require disentanglement
- Consideration should be given for call back, video from scene and other modalities (such as location finding) to enhance the fidelity of triage response
- During the initial call all casualties should be asked to self-extricate if able from their vehicles
- During the initial call casualties with neck and / or back pain should be asked to self-extricate if able from their vehicles
- During the initial call casualties with lower limb injuries should be asked to self-extricate if able from their vehicles
- During the initial call casualties regardless of their injuries should be asked to self-extricate if able from their vehicles
- Collisions identified at 999 call as potentially requiring disentanglement should trigger a senior FRS extrication response
- Collisions identified at 999 call as potentially requiring disentanglement should trigger an expert FRS extrication response
- Collisions identified at 999 call as potentially requiring disentanglement should trigger a ‘enhanced’ clinical care response

- Collisions identified at 999 call as potentially requiring disentanglement should trigger a 'critical-care' clinical care response
- All MVCs (regardless of entrapment status) should warrant an immediate response triage category for prehospital medical services
- MVC with suspected entrapment should warrant an immediate response triage category for prehospital medical services
- A standard multi-agency MVC trauma message should be developed to ensure the correct resources are deployed.

Section 9: Audit standards and research

Pan/multi professional audit standards have not yet been developed.

TARN collects entrapment status - this is variably completed and limited to trapped / not-trapped [11].

The government report national extrication data (type and time) - this cannot currently be linked to casualty injury or outcome data [53].

Statements: Audit standards and research

- FRS training in clinical care for entrapped casualties should be standardised
- Multi-professional datasets should be developed to enable research and audit
- Multi-professional datasets should include casualty entrapment status
- Multi-professional datasets should include how unwell and or time-critical entrapped casualties
- Multi-professional datasets should include different extrication approaches and their variants
- Multi-professional datasets should include entrapment time
- Multi-professional datasets should include in-car casualty care and its timing
- Multi-professional audit standards should be developed to improve quality of casualty care and extrication practice

Selected References:

1. Mahoney PF, Carney CJ. Entrapment, extrication and immobilization. Eur J Emerg Med. 1996;3:244–6.

2. Czajkowski J, Kidd S. Extrication challenges. 10 tips for EMS crews working at extrication scenes. EMS on scene. JEMS. 2001;26:50–61.

3. Politis J, Dailey M. Extrication Fundamentals Proper care of the entrapped patient. JEMS. 2010;35:41–7.

4. Methner P. Extrication scene tips for EMS personnel: EMS considerations to enhance scene operations and personal safety. JEMS. 2004;29:28–30, 32, 34–5.

5. Abrams JI, Pretto EA, Angus D, Safar P. Guidelines for rescue training of the lay public. Prehosp Disaster Med. 1993;8:151–6.

6. Funk DL, Politis JF, McErlean M, Dickinson ET. Necessity of fire department response to the scene of motor vehicle crashes. Am J Emerg Med. 2002;20:580–2.

7. Fattah S, Johnsen AS, Andersen JE, Vigerust T, Olsen T, Rehn M. Rapid extrication of entrapped victims in motor vehicle wreckage using a Norwegian chain method – cross-sectional and feasibility study. Bmc Emerg Medicine. 2014;14:14–14.

8. Leotsakos A, Zheng H, Croteau R, Loeb JM, Sherman H, Hoffman C, et al. Standardization in patient safety: the WHO High 5s project. Int J Qual Health C. 2014;26:109–16.

9. JESIP. JESIP Joint Doctrine Guide [Internet]. 2021 [cited 2021 Dec 9]. Available from: https://www.jesip.org.uk/joint-doctrine

10. WHO. Global status report on road safety 2015. 2015; Available from: https://apps.who.int/iris/handle/10665/189242

11. Nutbeam T, Fenwick R, Smith J, Bouamra O, Wallis L, Stassen W. A comparison of the demographics, injury patterns and outcome data for patients injured in motor vehicle collisions who are trapped compared to those patients who are not trapped. Scand J Trauma Resusc Emerg Medicine. 2021;29:17.

12. Dunbar I. Vehicle Extrication – The Next Generation. LUKAS; 2021.

13. NDFFAEM. Road Traffic Accident Handbook. 2009.

14. Benger J, Blackham J. Why do we put cervical collars on conscious trauma patients? Scand J Trauma Resusc Emerg Med. 2009;17:44.

15. Aebli N, Rüegg TB, Wicki AG, Petrou N, Krebs J. Predicting the risk and severity of acute spinal cord injury after a minor trauma to the cervical spine. Spine J. 2013;13:597–604.

16. Nutbeam T, Fenwick R, Hobson C, Holland V, Palmer M. Extrication time prediction tool. Emerg Med

J. 2015;32:401.
17. Nutbeam T, Fenwick R, Hobson C, Holland V, Palmer M. The stages of extrication: a prospective study. Emerg Med J. 2013;31:1006–8.
18. Dixon M, O'Halloran J, Hannigan A, Keenan S, Cummins NM. Confirmation of suboptimal protocols in spinal immobilisation? Emerg Medicine J Emj. 2015;32:939–45.
19. Gabrieli A, Nardello F, Geronazzo M, Marchetti P, Liberto A, Arcozzi D, et al. Cervical Spine Motion During Vehicle Extrication of Healthy Volunteers. Prehosp Emerg Care. 2019;24:1–14.
20. Häske D, Schier L, Weerts JON, Groß B, Rittmann A, Grützner PA, et al. An explorative, biomechanical analysis of spine motion during out-of-hospital extrication procedures. Inj. 2020;51:185–92.
21. Nutbeam T, Fenwick R, May B, Stassen W, Smith J, Bowdler J, et al. Assessing Spinal Movement During Four Extrication Methods: A Biomechanical Study using Healthy Volunteers. 2021;
22. Nutbeam T, Fenwick R, May B, Stassen W, Smith JE, Wallis L, et al. The role of cervical collars and verbal instructions in minimising spinal movement during self-extrication following a motor vehicle collision - a biomechanical study using healthy volunteers. Scand J Trauma Resusc Emerg Medicine. 2021;29:108.
23. Nutbeam T, Kehoe A, Fenwick R, Smith J, Bouamra O, Wallis L, et al. Do Entrapment, Injuries, Outcomes and Potential for Self-extrication Vary with Age? A Pre-specified Analysis of the UK Trauma Registry (TARN). 2021;
24. Cowley A, Hague A, Durge N. Cervical spine immobilization during extrication of the awake patient. Eur J Emerg Med. 2017;24:158–61.
25. Sundstrøm T, Asbjørnsen H, Habiba S, Sunde GA, Wester K. Prehospital Use of Cervical Collars in Trauma Patients: A Critical Review. J Neurotraum. 2014;31:531–40.
26. Brown A, Low A. Self-extrication and selective spinal immobilisation in a polytrauma patient with spinal injuries. Trauma. 2020;22:229–32.
27. Fenwick R, Nutbeam T. Medical vs. true physical traffic collision entrapment. J Paramedic Pract. 2018;10:158–62.
28. Weninger P, Hertz H. Factors influencing the injury pattern and injury severity after high speed motor vehicle accident—A retrospective study. Resuscitation. 2007;75:35–41.
29. Palanca S, Taylor DM, Bailey M, Cameron PA. Mechanisms of motor vehicle accidents that predict major injury. Emergen Med. 2003;15:423–8.
30. Dias ARN, Abib S de CV, Poli-de-Figueiredo LF, Perfeito JAJ. Entrapped victims in motor vehicle collisions: characteristics and prehospital care in the city of São Paulo, Brazil. Clinics. 2011;66:21–5.

31. Conroy C, Tominaga GT, Erwin S, Pacyna S, Velky T, Kennedy F, et al. The influence of vehicle damage on injury severity of drivers in head-on motor vehicle crashes. Accid Anal Prev. 2008;40:1589–94.
32. Carr BG, Caplan JM, Pryor JP, Branas CC. A meta-analysis of prehospital care times for trauma. Prehosp Emerg Care. 2006;10:198–206.
33. Vincent-Lambert C, Mottershaw T. Views of emergency care providers about factors that extend on-scene time intervals. Afr J Emerg Medicine. 2018;8:1–5.
34. Wilmink AB, Samra GS, Watson LM, Wilson AW. Vehicle entrapment rescue and pre-hospital trauma care. Injury. 1996;27:21–5.
35. Augustine JJ. Priorities in extrication. Emerg Med Serv. 1994;23:53–6, 58–61.
36. Mason AM. Prehospital Use of the Intubating Laryngeal Mask Airway in Patients with Severe Polytrauma: A Case Series. Case Reports in Medicine [Internet]. 2009;2009:1–7. Available from: http://www.hindawi.com/journals/crim/2009/938531/
37. Grosomanidis V, Amaniti E, Pourzitaki C, Fyntanidou V, Mouratidis K, Vasilakos D. Comparison between intubation through ILMA and Airtraq, in different non-conventional patient positions: a manikin study. Emerg Med J. 2012;29:32–6.
38. Hulme J, Perkins GD. Critically injured patients, inaccessible airways, and laryngeal mask airways. Emerg Med J. 2005;22:742–4.
39. Connor D, Greaves I, Porter K, Bloch M, Care consensus group F of P-H. Pre-hospital spinal immobilisation: an initial consensus statement. Emerg Med J. 2013;30:1067–9.
40. Armstrong BP, Simpson HK, Crouch R, Deakin CD. Prehospital clearance of the cervical spine: does it need to be a pain in the neck? Emerg Med J. 2007;24:501–3.
41. Oteir AO, Smith K, Stoelwinder JU, Middleton J, Jennings PA. Should suspected cervical spinal cord injury be immobilised?: A systematic review. Injury [Internet]. 2015;46:528–35. Available from: http://dx.doi.org/10.1016/j.injury.2014.12.032
42. Holla M. Value of a rigid collar in addition to head blocks: a proof of principle study. Emerg Med J. 2012;29:104–7.
43. Hauswald M. A re-conceptualisation of acute spinal care. Emergency Medicine Journal [Internet]. 2013;30:720–3. Available from: http://emj.bmj.com/cgi/doi/10.1136/emermed-2012-201847
44. Abarbanell NR. Immobilization and extrication of trauma patients by untrained personnel. Am J Emerg Med. 1991;9:98–9.
45. Hauswald M, Ong G, Tandberg D, Omar Z. Out-of-hospital spinal immobilization: its effect on neurologic injury. Acad Emerg Med. 2008;5:214–9.

46. NFCC. National Operational Guidance [Internet]. National Operational Guidance. 2021 [cited 2021 Oct 21]. Available from: https://www.ukfrs.com/guidance/
47. Brown JB, Rosengart MR, Billiar TR, Peitzman AB, Sperry JL. Distance matters. J Trauma Acute Care. 2017;83:111–8.
48. Sampalis JS, Denis R, Fréchette P, Brown R, Fleiszer D, Mulder D. Direct transport to tertiary trauma centers versus transfer from lower level facilities: impact on mortality and morbidity among patients with major trauma. J Trauma. 1997;43:288–95; discussion 295-6.
49. Brown JB, Rosengart MR, Forsythe RM, Reynolds BR, Gestring ML, Hallinan WM, et al. Not all prehospital time is equal. J Trauma Acute Care. 2016;81:93–100.
50. Simon BJ, Legere P, Emhoff T, Fiallo VM, Garb J. Vehicular trauma triage by mechanism: avoidance of the unproductive evaluation. J Trauma. 1994;37:645–9.
51. C D, P D, C P, S S, M B, G S, et al. Predicting the Need for Extrication in Traffic Accidents Reported to 911: Is Anyone Pinned/Trapped? AEDR. 2018;6.
52. Lerner EB, Shah MN, Cushman JT, Swor RA, Guse CE, Brasel K, et al. Does Mechanism of Injury Predict Trauma Center Need? Prehosp Emerg Care. 2011;15:518–25.
53. Fenwick R, Nutbeam T, Dayson M. A 10-year time series analysis of roof removal extrications by Fire and Rescue Services in England. Faculty of Prehospital Care Scientific Conference. 2020;
